# Vision degrading myodesopsia assessed with optos ultra-widefield scanning laser ophthalmoscope

**DOI:** 10.1186/s12886-023-03166-y

**Published:** 2023-10-20

**Authors:** Tiezhu Lin, Cheng Shi, Bing Wu, Emmanuel Eric Pazo, Lijun Shen

**Affiliations:** 1 Ophthalmology Department, Zhejiang Provincial People’s Hospital, Hangzhou, Zhejiang Province China; 2 He Eye Specialist Hospital, Shenyang, Liaoning China; 3 Dalian Medical University, Dalian, Liaoning Province China

**Keywords:** Floaters, Optos, Vision degrading myodesopsia, Scanning laser ophthalmoscope

## Abstract

**Background:**

To investigate the diagnostic sensitivity of Optos imaging for vision degrading myodesopsia (VDM).

**Methods:**

A total of 420 eyes from 345 patients with VDM were collected in this cross-sectional study. All eyes were classified as having posterior vitreous detachment (PVD) or not having PVD. The sensitivity of Optos imaging for the visibility of vitreous floaters was evaluated. The associated factors with the visibility of vitreous floaters on Optos images were analyzed in univariate and multivariate logistic regression analyses.

**Results:**

The mean age of all patients was 56.19 ± 13.89 years old, and 66.67% of patients were female. The vitreous floaters were visible on the ultrasound B scan in all eyes, but only in 47.62% of Optos images (55.29% in eyes with PVD and 15% in eyes without PVD). In the multiple binary logistic regression analysis, age (OR = 1.094, 95%CI = 1.063–1.125, P < 0.001), spherical equivalent (OR = 0.869, 95%CI = 0.791–0.955, P = 0.004) and the distance of the floaters from the retina (OR = 1.191, 95%CI = 1.059–1.339, P = 0.003) were significantly correlated with the visibility of vitreous floaters on Optos images. On Optos images, 25.71% of VDM eyes presented additional retinal abnormalities.

**Conclusions:**

Optos imaging has a low sensitivity for vitreous floaters, particularly in eyes without PVD. On Optos imaging, floaters were more visible in older patients, eyes with greater myopia, and floaters that were further from the retina.

## Introduction

Vision degrading myodesopsia (VDM) affects an increasing number of adults due to the global prevalence of myopia and the aging of the population [1]. As the eyes move, VDM may manifest as spots, cobwebs, shadows, and other diverse forms [2, 3]. While floaters are typically only a minor annoyance, some eyes develop numerous symptomatic vitreous opacities that significantly impede reading, driving, and other daily visual activities. VDM can be diagnosed with a dilated fundus exam and/or an ultrasound B scan. Although the ultrasound B scan is regarded as the gold standard for diagnosis and evaluation of VDM [4, 5], other imaging devices such as optical coherence tomography (OCT) and scanning laser ophthalmoscope are being used for VDM [6]. Optos, an ultra-widefield imaging system utilizing a scanning laser ophthalmoscope, is capable of obtaining fundus images with a horizontal field of view of approximately 200 degrees without pupillary dilation [7]. Because eyes with acute floaters are at risk for retinal tears and detachment [8], Optos imaging is commonly used to assess the condition of vitreous and retina in VDM eyes. However, the sensitivity of Optos imaging to detect vitreous floaters is unknown. In this study, we will explore the sensitivity of Optos imaging for monitoring vitreous floaters in VDM and define the factors associated with the visibility of floaters on Optos images.

## Methods

### Patient selection

The institutional ethics committee of the He Eye Specialist Hospital gave its approval to this cross-sectional study in accordance with the Helsinki Declaration’s guidelines. Following informed consent, 420 eyes from 345 patients with VDM were included in this study, which was conducted between January 2022 and August 2022, with the participants recruited from the outpatient clinics of He Eye Specialist Hospital. All patients complained of floater symptoms in either one or both eyes. Eyes with secondary vitreous floaters, such as vitreous inflammation or hemorrhage, were excluded. Eyes with images of poor quality were also excluded.

### Ophthalmological examination

**Fig. 1:**
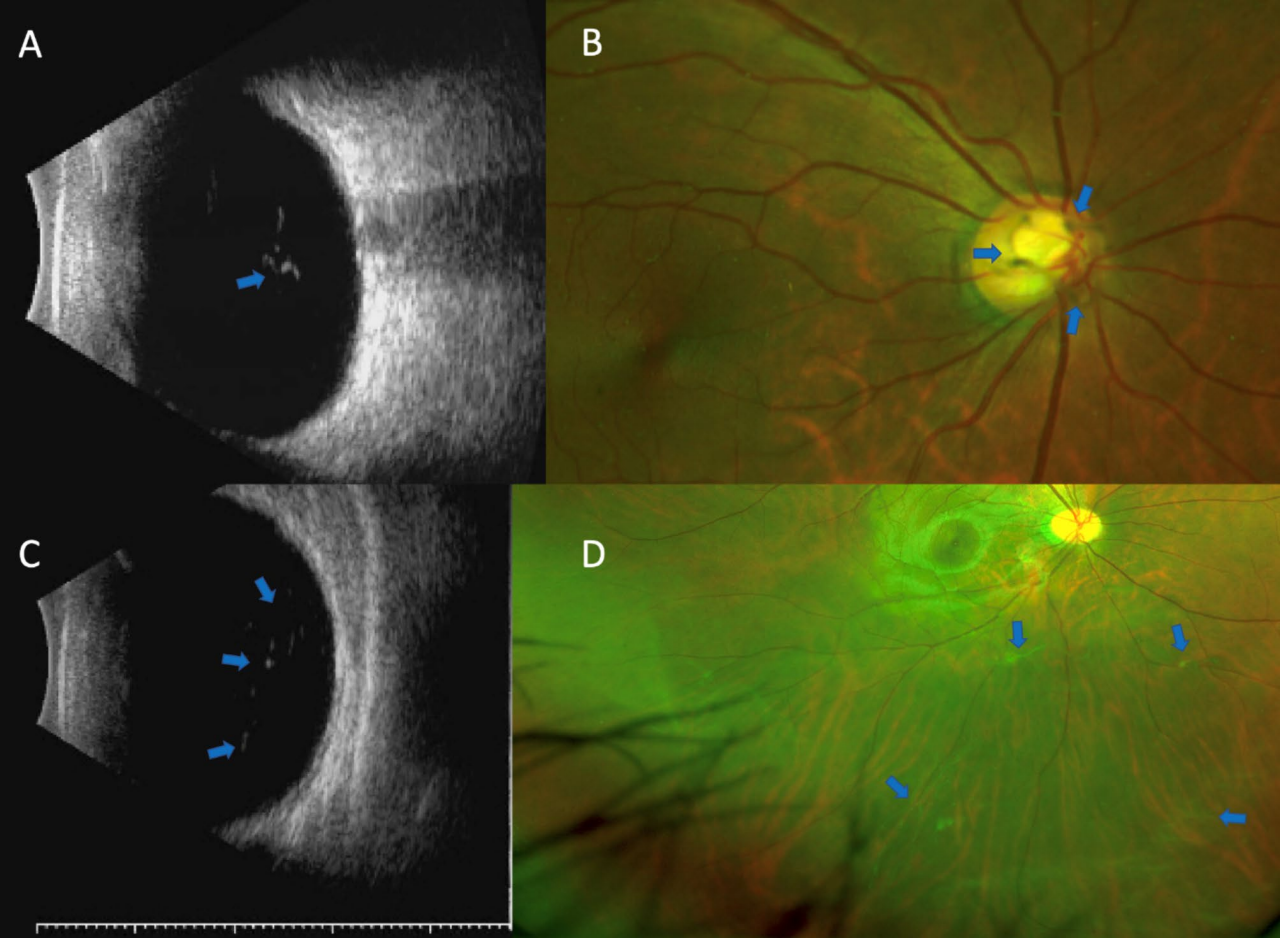
PVD noted on Ultrasound B scan and Optos images. The hyperreflective ring (blue arrow) in the vitreous cavity on the ultrasound B scan is suggestive of a Weiss ring (**A**), with the visible Weiss ring (blue arrow) noted on the Optos images (**B**). The hyperreflective line (blue arrow) in the vitreous cavity on the ultrasound B scan is suggestive of a detached hyaloid membrane (**C**), with the visible detached membrane (blue arrow) appearing on the Optos images (**D**)

Slit-lamp, visual acuity, intraocular pressure, refractive errors, vitreous and fundus examinations through a dilated pupil, ultrasound B scan (MD-2400 S, MEDA Co., Ltd, Tianjin, China), and Optos (Optos® 200Tx, Optos®, Dunfermline, U.K.) examinations were performed on all eyes. In the ocular ultrasound B scan, the vitreous status was determined through the eyelid contact method with gel with a 10 MHz probe. The gain of the B scan was set at 90 db. The mobility of the posterior vitreous was examined during saccadic eye movements using both vertical and horizontal views. The definition of vitreous floaters is hyperechoic material that is only visible in the vitreous cavity of the eye. A posterior vitreous detachment (PVD) status was considered when the posterior vitreous cortex was well defined and completely separated from the retina (Fig. 1) [9].

### Data collection

The baseline demographic data were collected, including age, gender, laterality of the involved eye, spherical equivalent, and duration of floater symptoms. On ultrasound B scan images, the shortest distance of floaters from the retina would be measured, and the highest reflective spots in the vitreous cavity would be chosen to measure. This distance was measured by two investigators (CS and BW), and the average value was used. The visibility of floaters on Optos images was also independently assessed by two investigators (CS and BW), and for the inconsistencies, the senior investigator (TZL) made a final decision.

### Statistical analysis

Continuous values were represented by the mean and standard deviation, whereas categorical variables were shown as percentages. Using the intraclass coefficient (ICC), the inter-rater agreement for the visibility of vitreous floaters on Optos images was determined. The agreement between reviewers was high: ICC = 0.971 (p < 0.001). Using univariate and multiple binary logistic regression analyses, risk factors associated with the visibility of vitreous floaters on Optos images were identified. For this study, the alpha level was set to 0.05, and the statistical power was established at 95%. All statistical analyses were performed with SPSS (SPSS Inc., USA), version 27.0.

## Results

In total, 420 eyes from 345 VDM patients were collected for this study. The mean age of all patients was 56.19 ± 13.89 years old, with a distinct female predominance (66.67%). There were 84 (20%) eyes with emmetropia, 144 (34.29%) eyes with low and moderate myopia, 36 (8.57%) eyes with high myopia, and 156 (37.14%) eyes with hyperopia. The major type of floaters was PVD (80.95%). The vitreous floaters were visible on Optos images in only 47.62% of eyes (55.29% in eyes with PVD and 15% in eyes without PVD) (Table 1).

**Table 1:** The demographic data of patients

	n (%)
**Age (yrs)**	
<30	25(7.25)
30–39	22(6.38)
40–49	32(9.28)
50–59	108(31.30)
60–69	121(35.07)
≥ 70	37(10.72)
**Gender**	
Male	115(33.33)
Female	230(66.67)
**Refractive errors**	
Myopia (≤-0.5D)	180(42.86)
Emmetropia (-0.5D~-0.5D)	84(20.00)
Hyperopia (≥ 0.5D)	156(37.14)
**SE(D) (mean ± SD)**	-1.22 ± 3.37
**Duration of floater symptoms (days) (mean ± SD)**	258.19 ± 1022.19
**The distance of the floaters from the retina (mean ± SD)**	4.38 ± 2.22
**Types of floaters**	
PVD	340(80.95)
Non-PVD	80(19.05)

SE = Spherical equivalent, PVD = posterior vitreous detachment

**Fig. 2:**
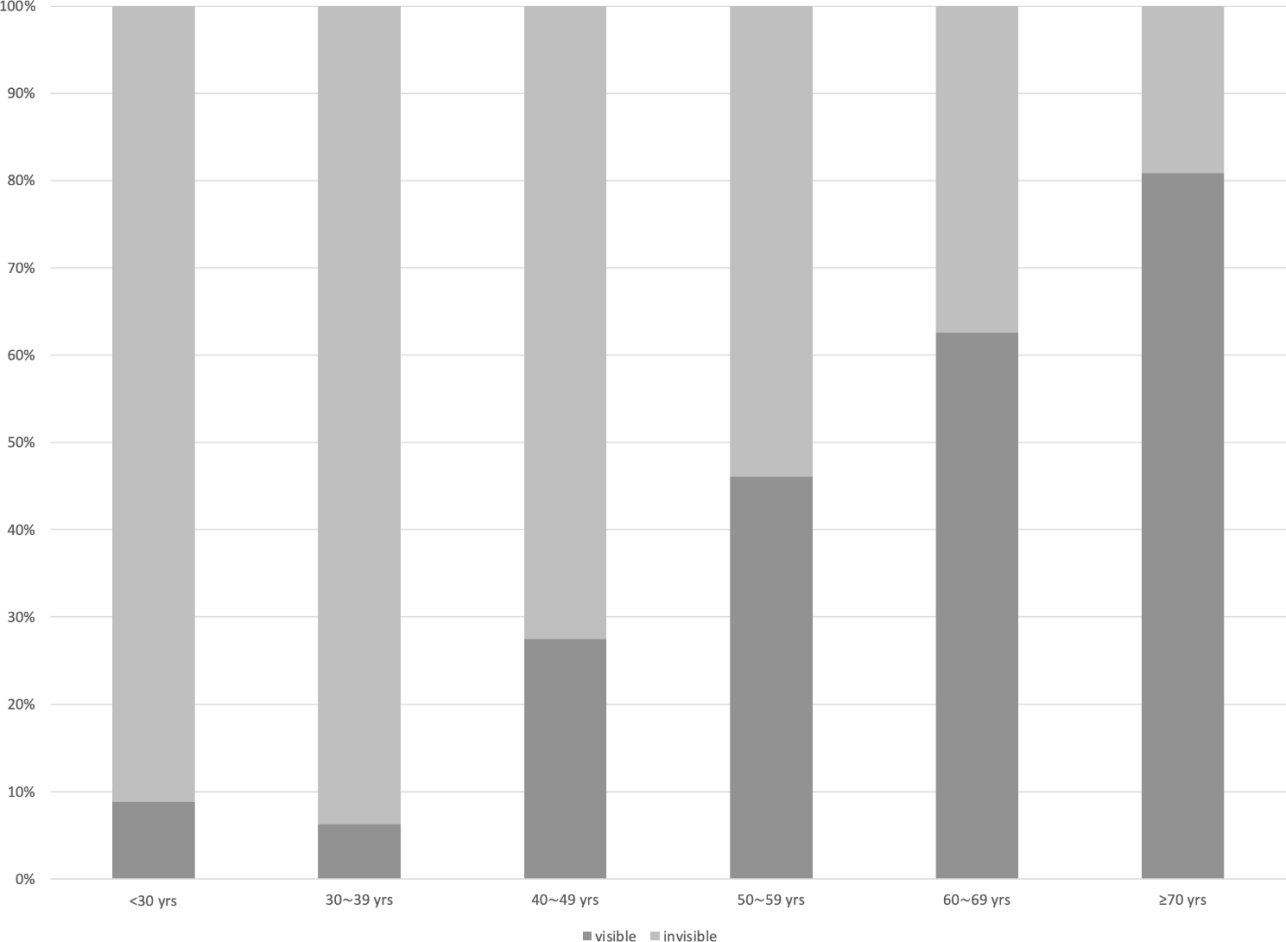
The sensitivity of Optos imaging for VDM with different age groups

**Fig. 3:**
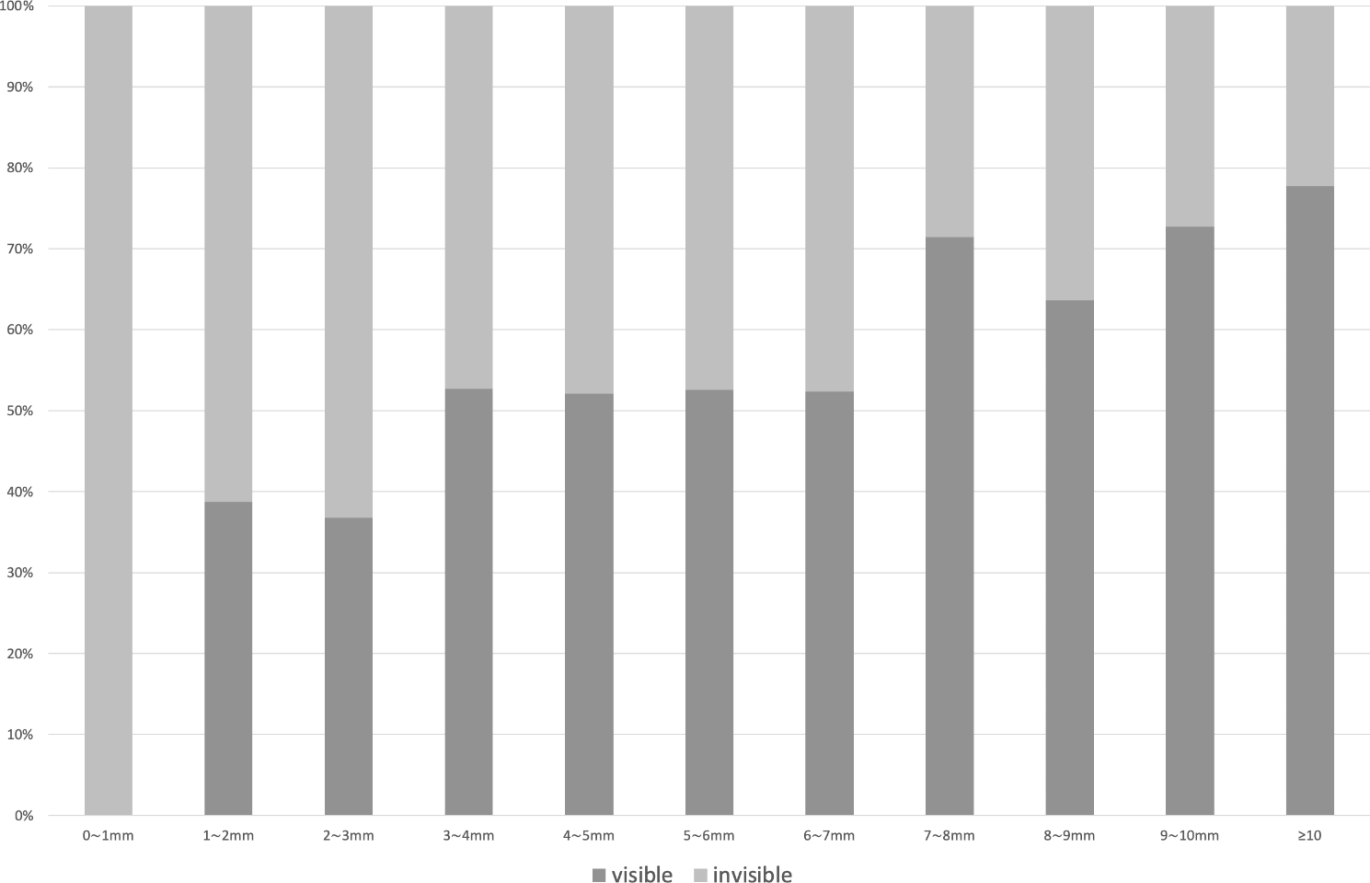
The sensitivity of Optos imaging for VDM with different floater distances

In the univariate analysis, age (OR = 1.083, 95%CI = 1.061–1.104, P < 0.001), the distance of the floaters from the retina (OR = 1.181, 95%CI = 1.069–1.304, P = 0.001), and floaters of PVD type (OR = 7.009, 95%CI = 3.659–13.424, P < 0.001) were significantly correlated with the visibility of vitreous floaters on Optos images. In the multivariate binary logistic regression analysis, age (OR = 1.094, 95%CI = 1.063–1.125, P < 0.001) (Fig. 2), spherical equivalent (OR = 0.869, 95%CI = 0.791–0.955, P = 0.004) and the distance of the floaters from the retina (OR = 1.191, 95%CI = 1.059–1.339, P = 0.003) (Fig. 3) were significantly correlated with the visibility of vitreous floaters on Optos images (Table 2).

**Table 2:** Univariate and multivariate logistic regression analysis for the risk factors associated with the visibility of floaters on Optos images

Variate	Univariate analysis		Multivariate analysis	
	OR (95%CI)	*P*	OR (95%CI)	*P*
Male	1.155(0.771–1.731)	0.485	1.565(0.925–2.648)	0.095
Age	1.083(1.061–1.104)	<0.001	1.094(1.063–1.125)	<0.001
Duration of floater symptoms	1.000(0.998–1.001)	0.486	1.000(0.998–1.001)	0.466
SE	1.009(0.953–1.068)	0.756	0.869(0.791–0.955)	0.004
The distance of the floaters from the retina	1.181(1.069–1.304)	0.001	1.191(1.059–1.339)	0.003
PVD	7.009(3.659–13.424)	<0.001	2.529(0.850–7.523)	0.095

OR = odds ratio, CI = confidence interval, SE = Spherical equivalent, PVD = posterior vitreous detachment

**Fig. 4:**
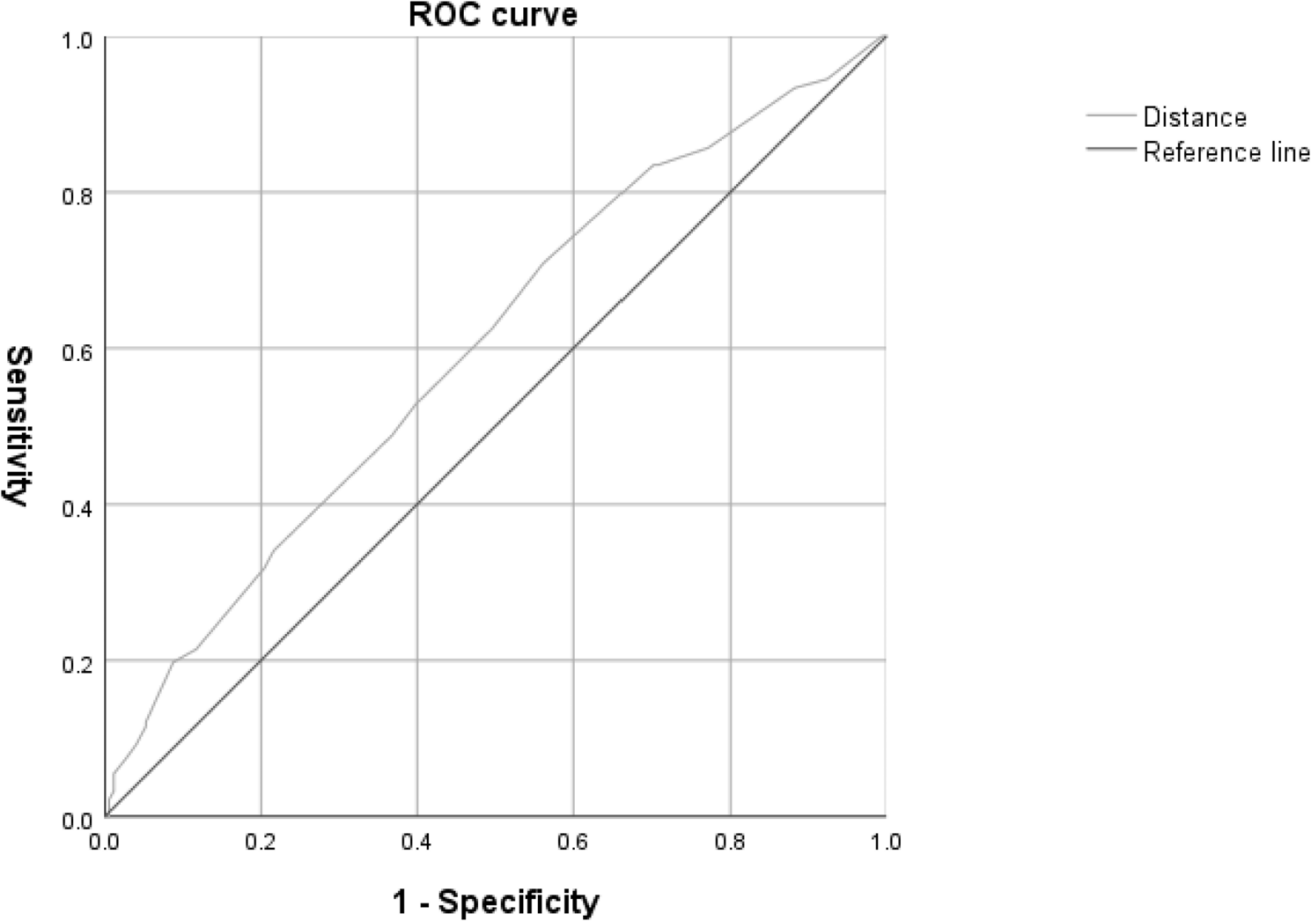
ROC analysis of the floater distance for the visibility on Optos images

The receiver operating characteristic (ROC) curve analysis of VDM patients with apparent floaters on Optos images revealed that the area under the curve of the distance of the floaters from the retina was 0.599 (Fig. 4). The optimum distance cut-off value for predicting floaters detected on Optos images was > 3.25 mm, which had a sensitivity of 71.04% and a specificity of 43.60%.

On Optos images, 25.71% of VDM eyes had additional retinal problems, such as drusen, epiretinal membrane, macular hole, peripheral retinal degeneration, choroidal nevus, Fuchs spot, diabetic retinopathy, bone-spicule-like pigmentation, and myelinated nerve fibers (Table 3).

**Table 3:** The other retinal abnormalities noted on Optos images

Other retinal signs noted on Optos	n(%)
**Drusen**	19(4.52)
**ERM**	10(2.38)
**MH**	1(0.24)
**Peripheral retinal degeneration**	33(7.86)
Lattice	4(0.95)
White without pressure	15(3.57)
Frost like	5(1.19)
Snail like	1(0.24)
Tears	8(1.90)
**Choroidal nevus**	2(0.48)
**Fuchs spot**	2(0.48)
**DR**	3(0.71)
**Bone-spicule like pigmentation**	1(0.24)
**Myelinated nerve fiber**	4(0.95)

ERM = epiretinal membrane, MH = macular hole, DR = diabetic retinopathy

## Discussion

VDM appears to be more prevalent among women, as reported in previous studies [10–14], which is consistent with the results of this study. Changes in hormones during perimenopause may affect how glycosaminoglycans are made and broken down [15, 16]. This could affect the vitreous collagen or the interface between the vitreous and the retina.

Ultrasound would be able to locate condensations deeper within the vitreous, ascertain their relationship to the central visual axis, and determine their mobility. Measuring the acoustic echo of reflected sound waves as they reach tissues of varying densities is fundamental to ultrasonography. In this study, a 10 MHz probe was used, which has better sensitivity and can be used to look at low-intensity scatterers, like those in the vitreous humor, that are hard to find with a higher-frequency probe [17]. Interestingly, an ultrasound sound B scan showed high echo signals in the vitreous cavity of all VDM eyes in the present study. Obviously, not every eye with vitreous echodensity would display VDM symptoms. Nguyen et al. [18] used quantitative ultrasonography to assess the vitreous structure; they also found vitreous echodensity in the eyes without floater symptoms. Hence, just using an ultrasound sound B scan to diagnose VDM should be oversensitive.

The Optos ultra-widefield imaging system is a scanning laser ophthalmoscope that can acquire widefield images at 532 nm (green) or 632 nm (red) scanning laser wavelengths. The two images may be viewed independently or superimposed to produce a semi-realistic color image. Compared to conventional imaging systems, the use of two laser wavelengths is advantageous because the red laser wavelength penetrates deeper into the retina and choroid, and the green laser wavelength produces better images of the retinal surface layers and retinal arteries. Optos imaging was reported to evaluate vitreous floaters with a sensitivity of 81.82% for PVD-type floaters and 58.62% for non-PVD-type floaters in a previous study, but that sample size was pretty small [12]. Son et al. [6] used Optos and optical coherence tomography to evaluate vitreous floaters, and peripapillary vitreous opacity was found in 62.2% of patients (122 out of 196 eyes). That result is comparable to the current study (55.29%). On Optos images, only 15% of eyes with non-PVD floaters were visible in the current study. The most frequent cause of non-PVD floaters is fibrillar aggregation, which can interfere with photon transmission sufficiently to cause long-lasting and progressive floaters [19, 20]. These floaters typically appear as strands or tiny dots [19, 20]. Optos imaging has difficulty projecting the shadows of these floaters on occasion. In addition, numerous variables, such as the floater’s size, density, and distance from the retina, were found to be correlated with the projection of floater shadows [21]. In this study, vitreous floaters further from the retina were easier to image on Optos imaging. This may be due to the fact that floaters nearer the laser’s light source cast larger shadows [21]. However, based on ROC, sensitivity, and specificity, the accuracy of distance from the retina in predicting the presence of images on Optos images was not good. In the current study, vitreous floaters in greater myopic eyes were also more visible on Optos imaging. We hypothesized that the higher degree of myopia may be accompanied by more severe vitreous liquefaction, resulting in more severe vitreous opacity, thereby increasing the visibility of vitreous floaters on Optos imaging [5]. Previous studies have demonstrated that as one ages, vitreous liquefaction worsens and vitreous collagen changes [5]. The loss of type IX collagen causes more type II collagen fibers to assemble into larger fiber polymers, which may increase the visibility of vitreous floaters on Optos images. In contrast, as the population ages, PVD will account for a greater proportion. Larger floaters, like a Weiss ring or a detached hyaloid, are also simpler to find [5]. In the current study, PVD-type floaters also had a tendency to be evident on Optos images.

Moreover, because OCT provides superior imaging of the vitreoretinal interface, numerous studies have employed it to evaluate VDM. Son et al. [6] identified variable degrees of PVD in 94.3% of eyes with new-onset floaters on OCT. The average onset period of floaters in their study was less than 10 days [6], which may explain the high sensitivity. The newly formed WEISS ring or detached posterior vitreous membrane was typically located near the retina and within the imaging range of OCT detection, whereas floaters farther from the retina are typically difficult to observe on OCT. Unfortunately, OCT was not used in this study. Infrared confocal scanning laser ophthalmoscopy (IRcSLO) has been used in recent studies to measure and grade the severity of floaters with high sensitivity [22–25]. Patients’ symptoms were found to correlate positively with the IRcSLO grade of floaters [22, 23]. Ngo et al. [24] believed that IRcSLO imaged vitreous abnormalities related to how patients perceive their own floaters more effectively than B-scan ultrasonography. This may be due to the fact that IRcSLO and ultrasound B-scan have distinct imaging orientations and that IRcSLO images are more intuitive and patient-friendly than ultrasound B-scan images. As with the ultrasound B scan, IRcSLO imaging is oversensitive for VDM; asymptomatic vitreous opacities have also been identified on IRcSLO imaging [22].

This study has a number of limitations. Although we hypothesized that the size and density of floaters would also affect how they appear on Optos imaging, there is currently no credible way to measure them accurately [26]. Total vitreous opacity may also affect the imaging of floaters on Optos, and quantitative ultrasound may be used to assess the total vitreous opacity in the future [4]. However, so far as we know, this is the largest study that shows how well Optos imaging can find vitreous floaters in VDM.

In summary, the sensitivity of Optos imaging for vitreous floaters is low, especially in non-PVD eyes, but it is a useful adjunct because it can detect numerous other retinal abnormalities that could otherwise be missed.

## Data Availability

The datasets used and/or analysed during the current study are available from the corresponding author on reasonable request.

## Abbreviations

VDM: Vision degrading myodesopsia
PVD: Posterior vitreous detachment
OR: Odds ratio
ICC: Intraclass coefficient
ROC: Receiver operating characteristic
IRcSLO: Infrared confocal scanning laser ophthalmoscopy
SE: Spherical equivalent
DR: Diabetic retinopathy
MH: Macular hole
ERM: Epiretinal membrane
